# Surgical Copper-Plates, for the Use of Practical Surgeons

**Published:** 1837-07

**Authors:** 


### Art. XII.

*Chirurgische Kupfertafeln, zum Gebrauch für practische Chirurgen.* Herausgegeben von Dr. Robert Froriep, Professor an der Universität Berlin, &c.—*Weimar*, 1836.

*Surgical Copper-plates, for the Use of Practical Surgeons.* Published by Dr. Robert Froriep, Professor in the University of Berlin, &c.—4to. *Weimar*, 1836.

This is a work of much labour and considerable merit. It contains an elaborate series of surgical illustrations, in part original, in part supplied by eminent surgeons, or compiled from their treatises. The choice of subjects it embraces, so far as it has proceeded, is judiciously made; and the accompanying delineations are faithful and graphic.

Endowed with an unwearied spirit of research, gifted with an eminent talent for design, and occupying a situation, as *prosector* at the Charité, where the most ample scope is afforded him for pathological investigation, the editor is qualified in a peculiar manner for such an undertaking as the one before us. It were superfluous to add our persuasion that, under such auspices, the work, when completed, will constitute a valuable source of reference for the practical surgeon.

Seventy parts have already made their appearance, each containing five copper-plate engravings and about thirty pages of explanatory text. We shall here give some account of the most interesting papers contained in the last four numbers, now before us.

Under the head of *Incarceratio interna*, a remarkable instance of ileus, caused by simple pressure of a diverticulum upon the bowel, is described. The description is elucidated by an annexed sketch of the state of parts on cadaveric inspection; and the whole forms a valuable addition to the important memoir of Professor Rokitanski in our last number.

When a diverticulum is adherent to the umbilicus, or to any other point within the abdominal cavity, it may then give rise to strangulation. For, if a portion of intestine happen to slip beneath, it will suffer constriction and become strangulated, as occurred in the cases which have been recorded by Sandifort and Eschricht. But that by the mere tension of a diverticulum, of which the extremity had become attached to the abdominal parietes, the portion of small intestine over which that passes can be contracted to such a degree, that the propulsion of its contents is no longer possible, and a fatal ileus the consequence, is a circumstance hitherto nondescript. A case of this kind, however, presented itself to Dr. Froriep in the Charité at Berlin. The subject was a young man, aged twenty years. He was somewhat suddenly attacked with urgent symptoms of inflammatory ileus, which proved fatal in about eight days. On examination after death, Dr. F. discovered that the ileus had been occasioned by the mechanical obstruction of a certain part of the canal of the small intestine, caused by the compression of a diverticulum attached to the free border of an intestinal fold; which latter, up to its mesenteric attachment, was flattened in shape. The coarctation of this fold of intestine lying between the mesentery and diverticulum, was to be explained only on the mode of attachment of the diverticulum to the abdominal wall. Since neither the mesentery behind, nor the diverticulum in front, were coherent, the portion of the alimentary canal intermediate was not put upon the stretch, and therefore could not be closed in that way.

It became a question in what way had the point of the diverticulum become thus attached to the abdominal parietes? Now, this may be resolved in one of two ways. In general, diverticula present a smooth, rounded-off, free extremity, without any string-like prolongation. Sometimes, again, a ligamentous cord proceeds from the diverticulum to the internal surface of the umbilicus, and is firmly united to the umbilical cicatrix. It has been long since ascertained that this cord-like appendix is simply the remains of the *vasa omphalo-mesaraica.* This mode of attachment of a diverticulum may be considered as a congenital malformation, the result of an interruption in the formative process. Here, of necessity, the cord is always identified with the navel. Or, secondly, we may conceive, the loose and floating round extremity of the diverticulum to have been attacked with inflammation, and the product of coagulable lymph thence resulting, to have determined its cohesion with some point in the peritoneum; as was really the fact in the first case mentioned by Eschricht. The case here recorded by Froriep is to be reckoned as one of a mixed nature, partaking of both the above conditions. There was, in all probability, a diverticulum present, to the end of which, as has been sometimes observed, a ligamentous cord was appended, one inch in length and formerly unconnected to any other part. The free extremity of this had by a process of inflammation, become at some former period firmly attached to the serous envelop of the abdominal walls.

The point of practical importance in the present enquiry is, that, independently of violent inflammation, of intussusception or incarceration, a fatal ileus can result in consequence simply of the flattening of a fold of intestine from the compression exercised by a tense ligamentous band stretching across it.

Plate 346 exhibits the delineation of a permanently contracted condition of the knee-joint, depending on a cause hitherto undescribed.

Before Dupuytren demonstrated that the retraction of the fingers whereby they were gradually bent in towards the palm of the hand, and fixed in that position, was the consequence of a gradual shortening of the palmar aponeurosis, it was believed by surgeons that this curvature of the fingers was occasioned by retraction of their flexor muscles; and the practice was to cut across the tendons, and to leave the patient with a hand just as impotent as before the operation, until Dupuytren succeeded in restoring the original freedom of motion to the hand, by simply dividing the integuments and immediately subjacent ligamentous bands, which pass from the palmar aponeurosis to the phalanges and skin covering the fingers.

From a survey of Dupuytren's researches on the subject, it occurred to Dr. R. Froriep that the same principle might with propriety be extended to other muscular contractions, to prove that the resulting deformity in different articulations did not proceed from the muscles, but from the aponeurotic envelopes of such joints; and, with this object in view, he studied particularly those instances of permanent contraction of the knee-joint in which the shortening of the limb could not be imputed to cicatrices or other organic changes in the parts constituting the articulation, or in its vicinity. Such cases are not rare. When they occur, it is commonly found that the leg forms a right angle with the thigh, and allows of no farther extension or flexion. The occasional cause of this variety of stiff-joint is, according to Dr. F., most frequently a carious ulcer on the back of the foot or upon the anterior aspect of the leg over the tibia; sometimes, however, it is a superficial sore in the vicinity of the ankle, or about the fibula, oftener chronic neuralgic affections of the lower extremities; on one occasion, it seemed to depend on a rebellious ulcer near the sacrum, and once on psoas abscess. A remarkable example of this kind occurred in a child labouring under *hydrorachitis congenita,* in whom both knee-joints were inflexibly retracted, without the presence of inflammation or any organic alteration in the contiguous parts. On every occasion the muscles leading to and from the part were scrutinized, yet no permanent contraction could ever be detected in the flexor muscles of the ham or leg; on the contrary, their fleshy bellies were soft and relaxed. But, on attempting to extend that portion of the *fascia lata* which encloses the popliteal space, it was found to be excessively strained and tense, and it was easily recognized that this tension was not prolonged either upwards or downwards to the muscles. Another alteration in the knee joint observed in some of these cases was an apparent acuminated form of the knee, which always presented itself on every forced extension of the limb, although quite the reverse might have been anticipated from that movement. On careful examination, it was observed that the soft parts upon the lower margin of the patella were drawn by the above motion somewhat inwards, whereby the knee-pan seemed to advance forwards, and so render the knee more pointed than natural. It was also seen that the skin and cellular tissue in the neighbourhood of the affected joint were not more firm or tense than usual, nor indeed changed in any respect; but that the mass of muscles belonging to the thigh and leg appeared less voluminous than natural, in consequence, no doubt, of the protracted inaction of the member.

Three cases are adduced in corroboration of the principles above enunciated. They clearly show that the contraction of the joint was to be ascribed neither to muscles nor tendons, but to some modification in the ordinary relations of the *fascia lata,* and its processes to the parts they surround. This change in the ordinary relations of that membranous investment without a coexisting organic alteration in its texture is to be attributed to a peculiarity existing in ligamentous tissues generally, and which has not heretofore met with the attention it deserves. This consists in their possessing more than any other texture the property of adapting themselves with exactitude to the parts which they enclose, although such a result should not at first sight be anticipated in consequence of their stiff and unyielding nature. It might be thought that the skin and cellular substance were endowed with this property in a more eminent degree than the above tissue, but such is not the fact. The skin is certainly capable of considerable extension, as is seen where the volume of parts embraced by it is augmented. It does not however contract in like proportion when the bulk of subjacent parts is diminished. Hence the skin, under these circumstances, as in persons who from being stout have become lean and emaciated, is found to be loose and wrinkled. On the other hand, the cellular substance is susceptible of contracting again upon the decrease in bulk of organs invested by it. It commonly, however, undergoes, during a long-continued process of enlargement in organs, not merely a simple extension, but becomes at the same time thickened, and more or less altered in its physical characters. With aponeurotic membranes these effects never occur. The aponeuroses mould themselves, as it were, in extent and form to every change which parts involved by them experience, and that without any mutation whatever in their structure, provided adequate time is allowed for their distention. On this principle, we may conceive, how a joint maintained for a long time in a state of quiescence in one uniform posture may become permanently fixed, and that without the supervention of pathological alteration in any part. For, the investing aponeurosis, which in virtue of its fibrous fasciculi retains its normal firmness and inelastic nature, adapts itself with much precision to the bent knee-joint; becoming ere long contracted on the concave and stretched out upon the convex side. It constitutes, moreover, a dense capsule, which acquires the form of the flexed articulation, and precludes the possibility of the contained parts assuming any other position.

There are two modes of treatment by which this deformity may be corrected; one, is to incise those portions of he aponeurosis which oppose extension in consequence of their contraction and firmness; the other mode requires us to modify the local condition of the aponeurotic membrane, by means of a sustained and gradually increasing extending force which shall subdue the preponderant power of the aponeurotic fibres. This done, they will, in virtue of their inherent pliability, accommodate themselves once more to the straight position of the limb. The first, or Dupuytren's method is indicated in the instance of permanent contraction of the palmar aponeurosis, where it is of importance to gain time, and where indeed a long-continued extension would be difficult and generally speaking impracticable. In the case of the knee-joint, however, extension can be well and beneficially applied. It is true, that by a simple and safe operation, namely, division of the intermuscular aponeurosis at the lower extremity of the *linea aspera* of the femur, the limb can be straightened. Yet, experience has proved that the same end can be effectually accomplished by properly adapted mechanical contrivances, (as the apparatus of Lafond,) at the expense of a little time. When the limb has been thus brought into the straight position, passive motion is to be enjoined, to restore suppleness to the joint.

In the 70th Number, we find also a description of a *novel* variety of palmar contraction of the fingers.

The author allows that the ordinary efficient cause of contractions of the fingers assigned by Dupuytren, namely, "cicatrices in the skin of the volar surfaces of the fingers," is not only admissible, but highly appropriate where the cicatrix is of considerable extent, and supplies the place of lost skin along the whole internal surface of a finger; as occurs after burns and contused wounds. The author finds it difficult, however, to explain those cases, by no means rare, wherein, after a mere simple incised wound in the cutaneous integument of the flexor aspect of a finger, which heals by the first intention, leaving a faint linear scar, permanent incurvation of the finger ensues. The difficulty is commonly got quit of by referring it to division of the flexor tendon; that position is, however, untenable, when the lesion is confined to the surface, and the energy of the flexors is preserved; as demonstrable by the circumstance of the distorted finger still retaining its prehensile power. Moreover, if such incurvation be regarded as the consequence of section and functional interruption of the flexor tendons, it is plainly incurable, and ought to be let alone. If, again, it be ascertained that the tendon is unimpaired, it may naturally be asked, 1st, how comes it that incurvation should follow a simple cut on the palmar aspect of a finger? and, secondly, how can such incurvation be remedied?

While the author was endeavouring to solve these questions, an opportunity was afforded him, in the course of last summer, for their practical investigation. A young female died in the Berlin hospital, having an incurvation of the little finger of the left hand. The curvature was such, that the finger permitted only partial extension, while it could be freely bent without difficulty. On forcible extension being made, the soft parts constituting the first phalanx assumed a stringy aspect, (as in palmar contraction;) on carefully examining the surface of the finger, the dorsal aspect appeared normal, but on the flexor integument a fine scar was perceptible, running obliquely from within outwards from the second to the beginning of the first phalanx.

The author gives the following results of his researches.

"(*a.*) That the cicatrix expanded itself under the delicate cutaneous investment, and occupied the place of the adipose *cushion* of the first phalanx; but at the same time was intimately incorporated with the enveloping tendinous fibres of the *ligamentum palmare longitudinale* on both sides of that *cushion.* (*b.*) That the *ligamentum palmare longitudinale* itself, from the anterior margin of the proper palmar aponeurosis, was greatly, and in the vicinity of the cicatrix more than doubly, thickened, notwithstanding the tendinous longitudinal fibres were normal, between which the grey transparent texture of the cicatrix was apparent. (*c.*) That the contraction of this thickened palmar *cord* operated precisely as in the ordinary palmar contraction described by Dupuytren, but, owing to the relaxation of the proper palmar aponeurosis, in a less eminent degree; that from the cicatrix, and from the ulnar margin of the condensed palmar cord, tendinous lines of the nature of cicatrix proceeded backwards to the ulnar side of the first finger-joint, continuously as far as the *aponeurosis manus,* where it covers the abductor minimi digiti, and at the same time connected with the lateral digital aponeurosis. (*d.*) That, lastly, the fore-mentioned lateral digital aponeurosis (the continuation of the *aponeurosis manus,*) was equally thickened, elevated and shortened by the substance of the cicatrix, and by itself capable of effecting a moderate incurvation of the finger."

Hence the author establishes the fact, from elaborate dissection, that, in this case, there was indeed a palmar contraction present; not however dependent on contusion or pressure, as maintained by Dupuytren, but on inflammatory irritation consecutive to incision. From this it follows that the answer to the second question, or the suitable means of cure, is the division of the palmar cord; an operation first proposed by the above-named surgeon. The mechanism of the contraction is fully displayed in the figures of Plate CCCLVI., to which the reader is referred.

The 69th and 70th Numbers of Dr. Froriep's work contain some important observations upon "Induration of the *tunica vaginalis testis.*" It is a well known fact, that simple affections of this nature have been mistaken for those of a malignant type, by surgeons of repute. Hence, grave operations have been resorted to, where a mild treatment would have sufficed. Delpech, in particular, sanctioned the error; and Dr. Froriep has been at great pains to show the fallacy of the views entertained by that eminent writer concerning this subject. An excellent opportunity was afforded, a few years back, of investigating the true pathological condition of the parts in a case where castration had been (*needlessly*) performed by a distinguished surgeon of another country. In this case, the author found the glandular substance of the testicle perfectly sound, the tunica albuginea unaltered, but having upon it a stratum of a white firm substance, of from one to one and a half lines thick, enveloping the entire testicle. No further examination was instituted.

Since that occasion, the author has directed his attention to similar cases, and at length ascertained that chronic irritation, associated with the distention caused by a hydrocele of long standing, gives rise to three forms of exudation *in the substance*, or what is commonly designated *the external surface* of the *tunica vaginalis propria testis*, which is applied to the *albuginea*. The lymph becoming organized, constitutes three different varieties of thickening. When chronic irritation produces an exudation of lymph and consequent augmentation in volume of the original textures, this is usually termed, (as, for example, in the cellular tissue,) *induration;* and with this meaning affixed to it, the author proposes to indicate thereby, the following varieties of alteration in the tunica vaginalis: 1. Induration of the tunica vaginalis reflexa alone, or the ordinary form of condensation of the membrane lying beneath the tunica vaginalis and cremaster. 2. Induration of the tunica vaginalis inversa testis, or exudation betwixt the tunica vaginalis and albuginea. 3. Induration of the entire tunica vaginalis (both *reflexa* and *inversa*), or condensation thereof, as well beneath the tunica vaginalis communis and cremaster, as in the albuginea testis. The last two of these forms, not heretofore distinctively described, are illustrated in the work before us, by cases and coloured plates.

1. Condensation of the *tunica vaginalis reflexa* alone is remarkably frequent, but seldom suspected to be present. An analogous thickening is always observed in serous membranes, when a portion has for a long time sustained considerable pressure from within outwards. This is seen in old herniæ, that have either not been returned for a great length of time or are wholly irreducible. Such hernial sacs, as well as the coverings of old large hydroceles, offer a compact opaque investment, of from one to two lines in thickness, having a leather-like consistence, not susceptible of being folded, but remaining tense and elastic, like a piece of parchment. It may be compared to a cartilaginous sheath, or to a bit of thin gum-elastic. The interior surface is, for the most part, quite smooth, nowise different from a healthy serous membrane: it is sometimes, however, uneven, presenting traces of plastic exudation and false membrane, the result of some acute inflammatory attack. Upon its external or cellular surface lies the cremaster muscle, just as in the healthy state of the parts: generally speaking, indeed, the tunica vaginalis communis, together with the cremaster, can be readily detached from the condensed serous membrane, unchanged throughout their extent. In certain old and large herniæ, the author has found the tunica vaginalis communis much thickened, and hardly separable from the indurated serous tunic.

But this is an exception, occurring only in old herniæ, never in hydrocele. Since, therefore, the *tunica vaginalis communis* can with so much ease be disunited from the thickened *tunica vaginalis testis reflexa*, the author is led to conclude that the condensation is seated in the delicate cellular tissue external to the serous surface; an opinion confirmed by microscopic examination. The whole of this indurated investing material, often from two to three lines thick, is formed by irregular granular inflammatory exudation, deposited in a mass of cellular *fibrous* bundles, exhibiting the same disposition as the *fibres* in the normal serous membranes, namely, an interlacement of undulating lines. This contexture is seen, under the microscope, to be altogether different from that of cartilage. In these condensations of the *tunica vaginalis reflexa*, the surface of the testicle is smooth; its serous envelope transmits the reflexion of the bluish albuginea; neither in its form, volume, or consistence, is ought unusual. Yet in certain cases the testicle is somewhat lax, chiefly in aged subjects long affected with hydrocele. In two instances of hydrocele in young persons, the testicle was firm and elastic, although the accumulation of fluid was considerable, and, in one of the two, of more than four years' duration. Should this thickened state of the investing membrane interfere with reunion by the first intention after the operation for hydrocele, it will be expedient to excise a portion of the morbid tissue.

II. The thickened condition of the *tunica vaginalis inversa* alone is more rare than the preceding variety, and corresponds to what Roux has termed "scirrhus albugineæ." Betwixt the *tunica vaginalis testis inversa* and the *tunica albuginea* a layer of firm fibrous substance is discovered, variable in thickness, united to, but easily separable from, the serous tunic. The main point in reference to practice connected with this form of induration is the diagnosis between it and the proper degeneration of the testicle. Nor is this difficult; since it may be affirmed that, whenever the latter feels elastic and smooth, so that its integral parts can be made to glide, as it were, one upon the other, the glandular substance may be reckoned perfectly normal, and it can be felt without difficulty even through the thickened stratum: whereas, in fungus medullaris, even before the disease has gone far, the testis is always unduly hard; a fact pointed out by Sir A. Cooper.

III. Induration of the whole tunica vaginalis testis, or thickening thereof, both beneath the tunica vaginalis testis and over the albuginea, presents two varieties: the one is distinguished by the testicle remaining free in the cavity of the tunica vaginalis, while it is covered with those knotty protuberances formerly noticed under the second form, and the tunica vaginalis reflexa is thickened in precisely the same way as in the first. In this variety the thickened stratum is chiefly beneath the serous surface, more especially at the situation of transition of the *tunica vaginalis reflexa* into the *inversa*, or *ligamentum epididymidis*, as it has been named, it is further prolonged into the cellular texture appertaining to the other testicle. The second variety has been already noticed by Baillie and Delpech. Here the testicle does not protrude within the tunica vaginalis, being prevented by the quantity of adventitious deposit. The testicle, from the pressure of fluid, is broader and flatter than natural, and is found lying on the posterior inferior side of the tunica vaginalis, when that is opened into. The condensation of that membrane is more marked here than in the foregoing variety: it does not, however, impede our recognizing by the finger the smooth elastic testicle.

From what has been stated, it is demonstrably evident that no operative interference is required for the relief of these indurative states of the tunica vaginalis. On the contrary, each of them ought to be treated as a simple hydrocele, and on no account whatever is extirpation of the testicle to be performed. Experience has proved that, after evacuation of the fluid, the morbid thickening of the tunica vaginalis gradually, and of its own accord, disappears. Any one who has carefully handled the healthy testis denuded (in the dead body,) will never be at a loss to recognize its normal consistence, and thereby remain possessed of a sure criterion for distinguishing a sound from a diseased one, however thickened the surrounding envelopes may be. All these points are well illustrated in the well-executed coloured plates accompanying the author's description.

---

